# Rethinking “Control” in Micro- and Nanoplastic Toxicology: Toward Exposure-Defined Reference States

**DOI:** 10.1021/acs.est.6c05960

**Published:** 2026-06-03

**Authors:** V. C. Shruti, Gurusamy Kutralam-Muniasamy

**Affiliations:** † Lab 49, Contaminantes Emergentes, Department of Biotechnology and Bioengineering, Centro de Investigación y de Estudios Avanzados del Instituto Politécnico Nacional, Av Instituto Politécnico Nacional 2508, San Pedro Zacatenco, Gustavo A. Madero, 07360 Ciudad de México, Mexico; ‡ 42576 CIITEC - IPN, Centro de Investigación e Innovación Tecnológica, Cda. de Cecati s/n, Santa Catarina, Azcapotzalco, 02250 Ciudad de México, Mexico

**Keywords:** control groups, toxicology, treatment effects, dose−response relationships

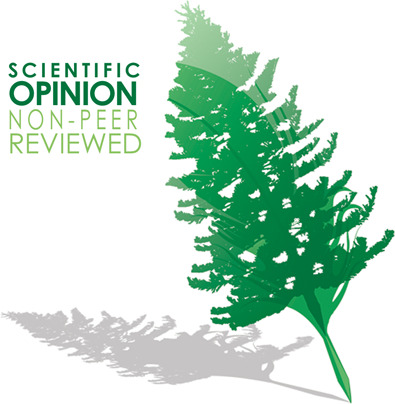

## Graphical abstract

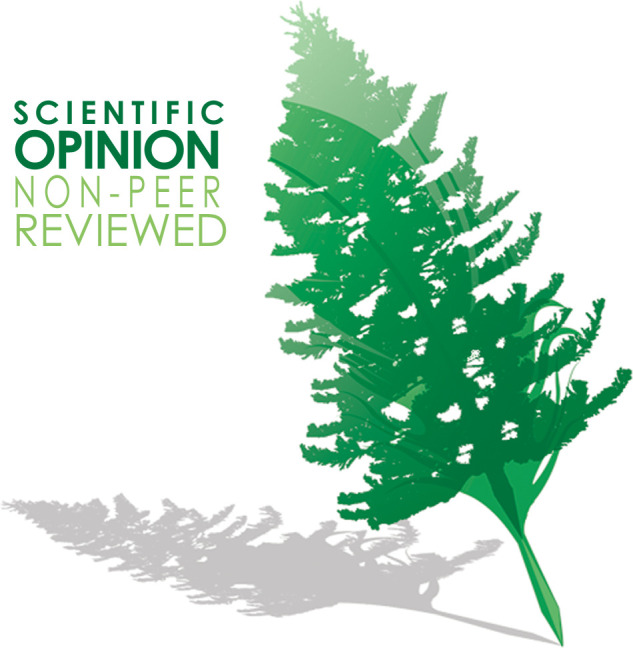

## Problem: Controls Are Not Zero-Exposure Systems

In micro- and nanoplastic (MNP) toxicology, control groups are conventionally treated as zero-exposure conditions against which treatment effects are evaluated, an assumption that underpins interpretation of dose–response relationships. Yet growing evidence suggests that organisms maintained under standard laboratory conditions can acquire MNPs through unavoidable background exposure.

The central issue is not contamination itself, which is already recognized within quality assurance and quality control (QA/QC) frameworks, but how control conditions are interpreted. Although procedural blanks, environmental monitoring, and negative controls remain essential for assessing external contamination and analytical reliability, they do not establish whether organisms themselves are free of internal exposure. Consequently, control organisms may continue to be interpreted as exposure-free reference states even when measurable internal particle burdens are present.

This disconnect creates a structural mismatch between environmental cleanliness and biological state. When control organisms carry measurable particle loads, the toxicological baseline becomes less certain, raising the possibility that low-dose responses reflect further perturbation of already exposed systems rather than effects arising in truly unexposed conditions.

## Empirical Basis: Background Exposure Is Biologically Realized

Evidence from laboratory systems suggests that background exposure is not incidental but persistent. Airborne deposition, waterborne contamination, and feed-associated particles have been documented under routine laboratory conditions, with deposition rates ranging from 4–82 particles per hour and procedural blanks containing 7–511 particles per sample.^1,2^ Together, these pathways indicate ongoing exposure potential even within controlled environments.

Importantly, these environmental signals appear to be biologically realized. Polymer residues detected in fecal samples of unexposed control rats (0.0306 g/day PA66; ∼ 1.2 mg/day mouse-equivalent) indicate ongoing ingestion and excretion under standard husbandry conditions, suggesting that background exposure becomes biologically incorporated rather than remaining solely an environmental signal.^3^

Evidence at the tissue level further supports this interpretation, with polyethylene particles reported in blood, liver, kidney, and endocrine tissues of control animals at concentrations reaching 11–70% of those observed in high-dose experimental groups.^4^ Such findings indicate that internal burdens in controls may overlap with experimental exposure ranges, complicating assumptions of an exposure-free baseline.

Despite these observations, most MNP toxicology studies do not routinely measure internal particle loads in control organisms. Instead, exposure status is often inferred from experimental design, leaving uncertainty in the biological reference state used for dose–response interpretation, particularly at environmentally relevant exposure levels.^5^

## Exposure-Defined Reference States

This raises a methodological question: what biological state does a control represent when exposure status is not empirically verified? We propose Exposure-Defined Reference States (EDRS) as a framework in which control conditions are defined through direct measurement of internal exposure, including particle burdens in tissues, feces, blood, or other biological matrices.

Although procedural blanks and environmental monitoring remain indispensable for identifying contamination pathways and ensuring analytical rigor, they characterize the exposure environment rather than the organism itself. The proposed framework therefore does not replace existing QA/QC systems but extends them by emphasizing characterization of the baseline internal burdens where feasible.

From this perspective, controls are no longer treated as presumed exposure-free states but as biologically characterized systems with measurable baseline burdens. The relevant distinction shifts the focus from presumed exposure status to empirically defined reference conditions.

## Operationalizing Control Conditions

Control conditions therefore exist along a continuum of biological characterization that moves beyond a strict exposed-versus-unexposed binary (Figure 1a).

**Figure 1:**
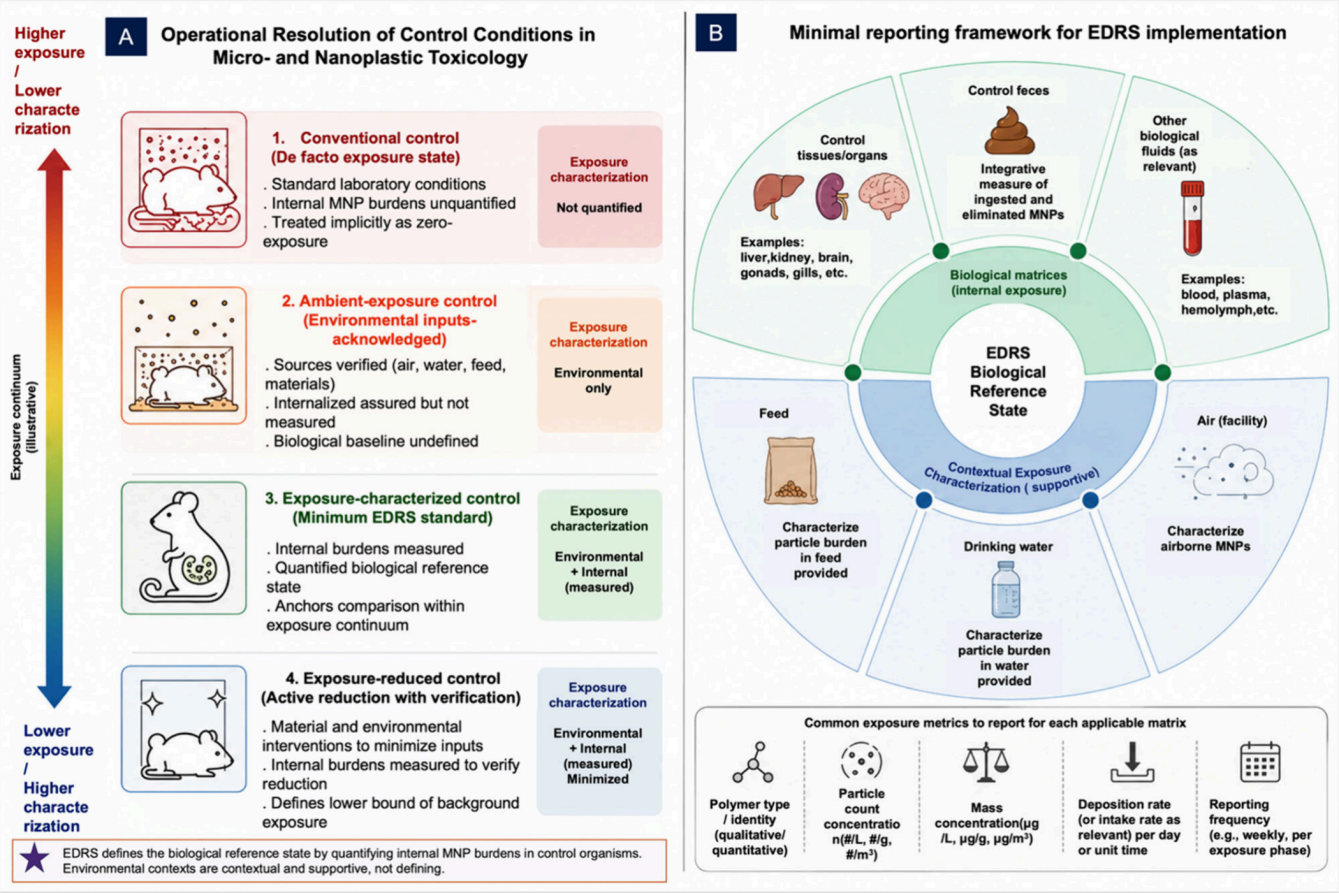
Exposure-defined reference states in micro- and nanoplastic toxicology. (A) Control conditions positioned along a continuum of baseline particle burden characterization and exposure reduction. (B) Recommended reporting structure for characterization of internal MNP burdens in control organisms, supported by contextual environmental measurements.

At one end, conventional controls rely on standard laboratory conditions without measurement of internal particle burdens, whereas ambient-exposure controls incorporate environmental monitoring but do not assess biological uptake. Exposure-characterized controls—the minimum EDRS-consistent standard—implement through empirical measurement of organismal particle loads across biological matrices. Exposure-reduced controls extend this approach further by pairing internal burden verification with procedural or environmental interventions designed to minimize dominant exposure pathways.

Within this framework, the interpretive value of a control depends less on an assumed absence of exposure than on how clearly its baseline exposure state has been characterized. Because appropriate matrices will vary according to organism type, particle type, and experimental end point, EDRS is not intended to impose a single methodological standard. Instead, the framework emphasizes transparent measurement and reporting of the biological state of control groups rather than reliance on environmental conditions alone.

Existing OECD and GLP frameworks already provide infrastructure for implementation of this approach. As outlined in Figure 1b, a minimum reporting framework can integrate environmental monitoring to contextualize exposure conditions while recognizing that such measurements cannot substitute for direct biological characterization of control organisms.

## Feasibility and Context Dependence

Implementation of EDRS will necessarily vary according to particle size, analytical sensitivity, and study design, such that nanoscale particles and chronic low-dose experiments may require greater characterization than larger microplastics or acute exposures. Although comprehensive quantification may not always be feasible, even partial characterization of internal burdens can improve interpretability when uncertainties in control exposure status are explicitly acknowledged (Figure 1b).

## Implications for Toxicological Inference

If control organisms carry measurable background burdens, dose–response relationships in MNP toxicology may compare systems with differing levels of preexisting exposure rather than truly exposed and unexposed states. In this context, variability across studies—particularly at environmentally relevant doses where baseline burdens may be biologically meaningful—may partly reflect differences in uncharacterized control conditions rather than inconsistencies in toxicological response. Direct measurement of internal burdens in controls could therefore strengthen cross-study comparability, improve meta-analytic interpretation, and help distinguish effects arising from new exposure, further accumulation on preexisting burdens, or interactions between the two.

As evidence for pervasive background exposure continues to accumulate, interpreting control groups as inherently exposure-free states becomes increasingly difficult to sustain. Reconceptualizing controls through EDRS shifts emphasis from assumed absence of exposure toward empirical characterization of biological baseline burdens. By clarifying the reference state underlying toxicological comparison, this framework strengthens interpretation while remaining compatible with existing experimental designs.
